# Analysis and prediction of relative survival trends in patients with non-Hodgkin lymphoma in the United States using a model-based period analysis method

**DOI:** 10.3389/fonc.2022.942122

**Published:** 2022-09-27

**Authors:** Shuping Xie, Zhong Yu, Aozi Feng, Shuai Zheng, Yunmei Li, You Zeng, Jun Lyu

**Affiliations:** ^1^ Department of Clinical Research, The First Affiliated Hospital of Jinan University, Guangzhou, China; ^2^ School of Basic Medicine and Public Health, Jinan University, Guangzhou, China; ^3^ School of Public Health, Shaanxi University of Chinese Medicine, Shaanxi, China; ^4^ Guangdong Provincial Key Laboratory of Traditional Chinese Medicine Informatization, Guangzhou, China

**Keywords:** non-Hodgkin’s lymphoma, relative survival rate, period analysis method, SEER, survival trend analyses

## Abstract

**Background:**

Survival rates are usually used to evaluate the effect of cancer treatment and prevention. This study aims to analyze the 5-year relative survival of non-Hodgkin lymphoma (NHL) in United States using population-based cancer registry data.

**Methods:**

A period analysis was used to evaluate the improvement in long-term prognosis of patients with NHL from 2004 to 2018, and a generalized linear model was developed to predict the 5-year relative survival rates of patients during 2019–2023 based on data from the SEER database stratified by age, sex, race and subtype.

**Results:**

In this study, relative survival improved for all NHL, although the extent of improvement varied by sex, age group and lymphoma subtype. Survival improvement was also noted for NHL subtypes, although the extent varied, with marginal-zone lymphoma having the highest 5-year relative survival rate (92.5%) followed by follicular lymphoma (91.6%) and chronic lymphocytic leukemia/small lymphocytic lymphoma (87.3%). Across all subtypes, survival rates were slightly higher in females than in males. Survival rates are lower in the elderly than in the young. Furthermore, the study demonstrated that black patients had lower NHL survival rates than white patients. Survival rates for NHL were higher in rural areas than in urban areas. Patients with extra-nodal NHL had a higher survival rate than patients with nodal NHL.

**Conclusion:**

Overall, patient survival rates for NHL gradually improved during 2004–2018. The trend continues with a survival rate of 75.2% for the period 2019–2023. Analysis by NHL subtype and subgroups indicating that etiology and risk factors may differ by subtype. Identification of population-specific prevention strategies and treatments for each subtype can be aided by understanding these variations.

## Introduction

Non-Hodgkin lymphoma (NHL) is a lymphohematopoietic system malignancy with a wide range of symptoms. NHL is the seventh most common cancer and the sixth leading cause of cancer death in the USA (1). It was estimated that 81,560 new cases of NHL and 20,720 patient deaths from NHL would occur in the USA in 2021 (2). Despite NHL being a prevalent cancer in children and adolescents, most NHL cases are in adults (3). Diffuse large B-cell lymphoma (DLBCL), chronic lymphocytic leukemia/small lymphocytic lymphoma (CLL/SLL), and follicular lymphoma (FL) are the three most common NHL subtypes, making up about two-thirds of NHLs. The most prevalent T-cell lymphoma subtype, known as peripheral T-cell lymphoma (PTCL), makes up about half of all T-cell NHLs (4).

NHL survival rates have recently improved due to advances in treatment methods (5). NHL incidence has decreased due to advancements in HIV/AIDS treatment (6). NHL prognosis differs with race, sex, and age. For example, NHL is most commonly diagnosed in adults older than 60 years, and younger patients have much higher survival rates (7). Some studies have found that the survival rates are in females than in males (8), and in white patients than in black patients (5). People with low socioeconomic statuses reportedly have lower survival rates after an NHL diagnosis (9).

Understanding the long-term survival trends of NHL and its prognostic factors could help with clinical prevention and treatment. Relative survival is a key indicator to assess patient prognosis. We used period analysis to stratify patients with NHL registered in the SEER database during 2004–2018 by age, sex, race, area, histology and disease site to assess their survival trends. We also developed a model-based period analysis method to predict the survival rate during 2019–2023 and explored possible causes of differences in survival with this period.

## Material and methods

### Data source

The data of this study were obtained from the SEER database, which is a large, population-based data set that collects cancer data from 17 registries across the USA and currently covers about 26.5% of that population. SEER*Stat software (version 8.4.0) was used to retrieve data for patients with NHL during 2000–2018 (10, 11). The follow-up information for patients was updated until December, 2019.

The inclusion criteria were (1) age >15 years and (2) NHL as the primary tumor. The exclusion criteria were (1) NHL diagnosis confirmed only by autopsy or death certificate, (2) incomplete data, (3) alive with no survival time, or (4) two or more primary malignant tumors.

### Data sorting

The WHO 2008 lymphoma classification system, which is based on ICD-O-3 site codes, was used to categorize NHL subtypes. The ICD-O-3 system codes for tumor site, histology, and malignant behavior. Some rare subtypes were excluded due to the low number of cases, which precluded analysis. Other indicators were classified according to the following criteria: sex (male and female), race (Non-Hispanic (NH) White, NH Black, NH American Indian/Alaskan Native, NH Asian and Pacific Islander), primary site (intra- and extranodal), and age (15–44, 45–54, 55–64,65–74, and 75+ years).

### Statistical analysis

Relative survival was used to evaluate patient prognosis. Relative survival is the ratio of the observed survival to the expected survival and is expressed as


$$R_{i}=\frac{\bar{S_{k}}}{S_{k}^{*}}$$

where $\bar{S_{k}}$ and $S_{k}^{*}$ represent the true and expected survival rates, respectively, and *k*=5 was used to calculate the 5-year relative survival rate.

Observed survival and expected survival were estimated using the life table method and the Ederer II method, respectively (12).The expected survival table was obtained from the Center for Disease Control and Prevention of the United States regularly published life tables for the population stratified by sex, individual years 1992 through 2018, individual ages from 0-99 years, by mutually exclusive race/ethnicity groups(Non-Hispanic (NH) White, NH Black, NH American Indian/Alaskan Native, NH Asian and Pacific Islander, and Hispanics), and by varied geography (13).

The 5-year relative survival rates of NHL patients assessed by the period analysis method in this study (2004-2018). The point estimates of relative survival and their standard errors were calculated using the Greenwood method. We classified patients into five major age groups (15–44, 45–54, 55–64,65–74, and 75+ years) according to the International Cancer Survival Standards (ICSS) for age standardization of survival.

A generalized linear model based on the period analysis method was developed to predict the 5-year relative survival rates of diagnosed patients from 2019-2023. The study first included cases diagnosed from 2004-2008, 2009-2013, and 2014-2018 according to the principles of the period analysis method. Finally, a regression model was fitted using the follow-up period and follow-up year as independent variables and the conditional 1-year survival rate for each year as the dependent variable. The above calculation and analysis process was performed using the “periodR” package in R software (14).

## Results

The SEER database 209257 patients with NHL diagnosed during 2000–2018. Among these patients, there were 112496 male patients and 96761 female patients. **Table 1** lists NHL cases that were confirmed and documented in the SEER database during each observation period. The most common NHL subtypes (DLBCL, FL, PTCL, MZL and CLL/SLL) accounted for more than three-quarters of the total NHL cases. White patients accounted for 68.9% of the cases. N-NHL was more common than EN-NHL regarding site, accounting for 66.0% of cases. The number of cases was relatively stable across observation periods regarding the distributions of sex, race, age.

**Table 1:** 2004–2018 Basic Situation of Non-Hodgkin lymphoma Incidence.

	2004-2008 (N=52228)	2009-2013 (N=56010)	2014-2018 (N=63194)
**Sex**			
Male	27682 (53.0%)	30312 (54.1%)	34587 (54.7%)
Female	24546 (47.0%)	25698 (45.9%)	28607 (45.3%)
**Race**			
American Indian/Alaska Native	238 (0.4%)	322 (0.6%)	326 (0.5%)
Asian or Pacific Islander	3402 (6.5%)	4223 (7.5%)	5385 (8.5%)
Black	3961 (7.6%)	4474 (8.0%)	4949 (7.8%)
White	37909 (72.6%)	38470(68.7%)	41657 (65.9%)
**Diagnosis age**			
15-44	7697 (14.7%)	7461 (13.3%)	7531(11.9%)
45-54	8160 (15.6%)	8425 (15.0%)	8299 (13.1%)
55-64	10497 (20.1%)	12442 (22.2%)	14535 (23.0%)
65-74	10636 (20.4%)	12527 (22.4%)	16336 (25.9%)
75+	15238 (29.2%)	15155 (27.1%)	16493 (26.1%)
**Primary Site**			
Extranodal	16529 (31.6%)	19283 (34.4%)	22511 (35.6%)
Nodal	35699(68.4%)	36727(65.6%)	40683 (64.4%)
**Area**			
Rural	45612(87.3%)	49625(88.6%)	56451(89.3%)
Urban	6563(12.6%)	6316(11.3%)	6697(10.6%)
**Histology**			
CLL/SLL	2923 (5.6%)	2435 (4.3%)	2843 (4.5%)
DLBCL	19379 (37.1%)	20953 (37.4%)	23708 (37.5%)
FL	9959 (19.1%)	9788(21.7%)	10919 (17.3%)
MCL	1937 (3.7%)	2359(4.2%)	2833 (4.5%)
MZL	4575 (8.8%)	5476 (9.8%)	6586(10.4%)
PTCL	3237 (6.2%)	3448 (6.2%)	3716 (5.9%)

**Table 2** lists the 5-year relative survival rates of NHL and its subtype by sex. According to the results of the period analysis, survival rates improved for both males and females during 2014–2018 when compared with 2004–2008, and females had better survival rates than males for all subtypes. Changes in relative survival varied by subtype. During 2014–2018, the 5-year relative survival rate was the highest for MZL (92.5%) followed by FL (91.6%) and CLL/SLL (87.3%). The 5-year relative survival rate increased for total NHL and NHL subtypes. The CLL/SLL had the best improvement, increased from 71.9% to 87.3% during 2004–2018. The generalized linear model predicts an increase in 5-year relative survival for NHL and its subtypes between 2019 and 2023.

**Table 2:** 5-Year relative survival rates for patients with non-Hodgkin’s lymphoma and its subtypes by sex from 2004 to 2018 and predicted relative survival rates for patients with non-Hodgkin’s lymphoma and its subtypes from 2019 to 2023.

		2004-2008	2009-2013	2014-2018	2019-2023
5 years survival rates	Sex				
NHL	Overall	63.5 ± 0.3	68.5 ± 0.2	73.3 ± 0.3	75.2
	Male	59.9 ± 0.4	65.8 ± 0.4	71.2 ± 0.4	73.1
	Female	67.6 ± 0.4	71.7 ± 0.3	76.1 ± 0.4	77.9
CLL/SLL	Overall	71.9 ± 1.1	80.3 ± 1.1	87.3 ± 1.3	90.9
	Male	67.5 ± 1.5	75.6 ± 1.5	86.2 ± 1.9	89.7
	Female	77.5 ± 1.5	86.2 ± 1.6	88.6 ± 2.0	91.7
DLBCL	Overall	54.1 ± 0.4	59.2 ± 0.4	63.4 ± 0.4	65.4
	Male	51.3 ± 0.6	57.6 ± 0.6	62.1 ± 0.6	64.7
	Female	57.5 ± 0.6	60.9 ± 0.6	65.1 ± 0.7	66.5
FL	Overall	80.7 ± 0.6	86.4 ± 0.6	91.6 ± 0.7	94
	Male	78.4 ± 0.9	85.3 ± 0.9	90.6 ± 1.0	93.2
	Female	82.7 ± 0.8	87.3 ± 0.8	92.2 ± 0.9	94.4
MCL	Overall	50.5 ± 1.3	58.4 ± 1.2	65.9 ± 1.4	66.3
	Male	48.6 ± 1.5	56.7 ± 1.4	64.1 ± 1.7	64.3
	Female	54.6 ± 2.3	62.0 ± 2.1	69.0 ± 2.4	69.9
PTCL	Overall	44.4 ± 1.1	50.5 ± 1.1	51.4 ± 1.2	52.8
	Male	40.5 ± 1.4	48.8 ± 1.4	49.5 ± 1.6	51.2
	Female	49.4 ± 1.6	52.8 ± 1.5	54.6 ± 1.8	55.5
MZL	Overall	88.2 ± 0.7	90.0 ± 0.7	92.5 ± 0.9	94.2
	Male	86.3 ± 1.3	87.9 ± 1.2	90.3 ± 1.5	92.1
	Female	89.7 ± 0.9	91.5 ± 0.8	93.9 ± 1.0	95.5

Survival rates is relative survival rates; data are means ± standard error of the mean.

The 5-year relative survival rates of NHL by age are listed in **Table 3**. In comparison with 2004–2008, the survival rate of patients in all age groups was improved during 2014–2018. The 5-year relative survival rate has been consistently higher in younger age groups compared to older age groups for NHL throughout the study period. In 2014-2018, the 5-year relative survival rate was 58.0% for the 75+ group, 75.8% for the 65-74 group, 80.0% for the 55-64 group, 84.2% for the 45-54 group, and 86.0% for the 15-44 group. The generalized linear model indicated that the highest 5-year relative survival rate for NHL was predicted for those aged 15–44 years at 89.2% during 2019–2023, while the lowest rate of 59.3% was predicted for those aged ≥75 years.

**Table 3:** 5-Year Relative Survival Rates of Non-Hodgkin lymphoma Patients by age group from 2004 to 2018 and Forecast of Non-Hodgkin Lymphoma Patients’ Relative Survival Rates from 2019 to 2023.

	2004-2008	2009-2013	2014-2018	2019-2023
5 years survival rates				
15-44	77.4 ± 0.5	83.3 ± 0.4	86.0 ± 0.5	89.2
45-54	76.1 ± 0.5	80.2 ± 0.5	84.2 ± 0.5	86.2
55-64	71.2 ± 0.5	76.1 ± 0.4	80.0 ± 0.4	81.3
65-74	64.0 ± 0.5	70.4 ± 0.5	75.8 ± 0.5	78.3
75+	48.2 ± 0.6	52.3 ± 0.6	58.0 ± 0.8	59.3

Survival rates is relative survival rates; data are means ± standard error of the mean.

**Table 4** lists the 5-year relative survival rates of patients with NHL by race. In 2014–2018, white patients had the highest survival rate of 75.7 ± 0.3%. The 5-year relative survival rates improved for patients of all races except American Indian/Alaska Native during 2014–2018 compared with 2004–2008. The generalized linear model predicted relative survival rates of 77.6%, 69.5%, 65%, and 67.6% for white, black, American Indian/Alaskan Native, and Asian or Pacific Islander patients, respectively, during 2019–2023. In addition, the survival of NHL was higher in rural areas than in urban (**Table 4**). The relative survival of NHL cancers in both urban and rural increased over time. Patients with extranodal NHL fared better than those with intranodal NHL regarding survival during 2014–2018 (**Table 4**). The relative survival of NHL cancers in both extranodal and nodal increased over time.

**Table 4:** 5-Year Relative Survival Rates of Non-Hodgkin lymphoma Patients by race, area and primary site from 2004 to 2018 and Forecast of Non-Hodgkin Lymphoma Patients’ Relative Survival Rates from 2019 to 2023.

	2004-2008	2009-2013	2014-2018	2019-2023
5 years survival rates				
White	65.6 ± 0.3	70.6 ± 0.3	75.7 ± 0.3	77.6
Black	53.5 ± 1.2	60.5 ± 1.1	65.6 ± 1.3	69.5
American Indian/Alaska Native	64.0 ± 4.3	67.2 ± 3.4	62.6 ± 4.5	65
Asian or Pacific Islander	58.7 ± 1.0	63.0 ± 0.9	66.0 ± 0.9	67.6
Rural	63.8 ± 0.3	68.8 ± 0.3	73.6 ± 0.3	75.6
Urban	61.3 ± 0.7	66.1 ± 0.7	70.7 ± 0.8	71.6
Extranodal	67.6 ± 0.5	72.5 ± 0.4	77.6 ± 0.5	80.6
Nodal	61.6 ± 0.3	66.5 ± 0.3	70.8 ± 0.4	72.1

Survival rates is relative survival rates; data are means ± standard error of the mean.

## Discussion

In this study, relative survival improved for all NHL, although the degree of improvement varied by sex, age group and subtype.

NHL is a malignancy that seriously threatens human health and life and can develop in all age groups with a high degree of heterogeneity and widely varying clinical features among different pathological subtypes (15). Males, people over 65, those with autoimmune disorders, and people who have a family history of hematological malignancies are more likely to develop NHL (16). Each subtype of NHL has a unique set of risk factors, making it a heterogeneous disease (12). There were more male than female patients, consistent with the 2018 GLOBOCAN data (17). The higher prevalence of certain risk factors (e.g., obesity (18), smoking (19), alcohol consumption (20), HIV (21), and chemical exposure in males (22)) may help explain why the incidence is higher in males than in females.

This study found that the relative survival rate of patients with NHL was increased during 2014–2018 compared with 2004–2008, and this trend continued during 2019–2023. It is assumed that these changes in survival rates reflect advances in the treatment of NHL (23). A notable advancement was the addition of new chemotherapeutic agents (rituximab) to standard chemotherapy (CHOP) since 1998. As rituximab became available, this initial regimen was used to treat the majority of DLBCL and FL cases (24, 25), which increased survival rates. In this study, between 2004 and 2018, survival rates increased from 54.1% to 63.4% for DLBCL and from 80.7% to 91.6% for FL. Fludarabine and rituximab were also introduced as new agents for the treatment of CLL/SLL in the 1990s (26). Because rituximab is only effective in B-cell NHL, outcomes in peripheral T-cell NHL remain poor due to the lack of therapeutic efficacy (27, 28).

It has been demonstrated that between 2000 and 2015, the incidence of Peripheral T-cell lymphoma increased, whereas the incidence of many other subtypes decreased (29). The increase in incidence is largely confined to stage IV. Clinical symptoms do not appear until advanced stages, making it challenging to detect and treat and giving the prognosis for Peripheral T-cell lymphoma the poorest of NHL subtypes. The discovery and implementation of improved stem cell transplantation techniques, new cytotoxic therapies, monoclonal antibodies such as rituximab, and most recently, targeted therapies have greatly improved the treatment of relapsed/refractory disease at all stages (30). Obesity and vitamin D deficiency worsen NHL survival (30). Obesity is associated with reduced survival in DLBCL and T-cell lymphoma (31).According to studies conducted in other nations, insufficient vitamin D has also been linked to lower survival rates for CLL/SLL (32), DLBCL, and T-cell lymphoma (33).

During 2014–2018, the highest survival rates for NHL were observed in younger patients. Survival rates improved over time among all disease stages and age groups, and were much higher in younger patients. Possible reasons for this are that older patients are less able to tolerate treatment (34) and their high risk of complications leads to inadequate treatment (35). Because optimum and curative treatments can only be given to individuals in whom the condition is detected sufficient early, and clinical staging is the most significant survival indicator (36). Chemotherapy cures most patients with limited lesions (Ann Arbor stage I/II), and patients with local lesions often have fewer adverse prognostic factors (22), resulting in higher survival rates when compared with patients with advanced lesions (stage III or IV). This study also found that females had higher relative survival rates than males, which was similar to the findings of Pulte et al. (8). The cause of the sex disparity is unknown, but it could be related to the different hormone levels and different immune function responses to treatments such as surgery between males and females (37).

Race is an important factor in the prognosis of NHL (38), and studies have found that incidence and survival rates are highest in white (39). The lower socioeconomic level of black relative to white patients may explain some of the disparities in the outcomes of patients with cancer (40). Certain racial disparities may be explained by other variables such as health-related behaviors, social support, and genetic vulnerability (41).For NHL, there has been progress in narrowing the survival gap between non-Hispanic white patients and ethnic minorities (42). Similarly, data on global socio-economic parameters of NHL showed large variations and higher mortality in low- and middle-income countries probably related to prevalence and underlying risk factors as well as access to medical care (43). Inequalities in socioeconomic level and health care access may influence the efficacy of therapy, and such disparities may impact NHL diagnoses (44). This is consistent with individuals having a low socioeconomic status being more likely to be diagnosed with advanced illness. Patients who receive chemotherapy have a reduced mortality risk (45). Enhancing early diagnoses for patients with low socioeconomic statuses may therefore help to reduce socioeconomic differences in NHL prognoses.

The prevalence of EN-NHL has increased more quickly than that of N-NHL over the past 20 years (46). This is partly due to the AIDS pandemic and improvements in diagnostic modalities (47). The present study found that N-NHL was more prevalent than EN-NHL. Intranodal primary sites were the most common in those with cervical lymph node enlargement, and extranodal primary sites were most often in the gastrointestinal tract, Wechsler’s ring, and nasal cavity (48). The proportional increase in EN-NHL may be linked to changes in living conditions, dietary habits, and increases in those with hepatitis B virus, HIV, EBV, and *H. pylori* infections (7, 49). In the present study, the prognosis of EN-NHL was found to be better than that of intranodal NHL, and the prognostic factors affecting it were mostly related to the degree of pathological malignancy, lesion location, lesion extent, and depth of tumor infiltration (50, 51). In another study, age, physical status, disease stage, and serum LDH levels were independent prognostic factors, while intra- or extranodal sites did not have any prognostic significance (52). Survival in EN-NHL has been found to depend on histological factors and disease stage (53). More patients with EN-NHL are diagnosed earlier than those with N-NHL; although EN-NHL differs from N-NHL, overall survival is largely dependent on IPI rather than the primary site of the malignancy (52). Survival rates did not differ between EN-NHL and N-NHL in early cases, while they were higher in N-NHL than EN-NHL patients in advanced cases (54).

## Limitations

There were limitations to this study: First, the SEER database tumor registry relies on provider diagnosis and documentation conditions, and if a disease is underdiagnosed or asymptomatic, as in the case of early-stage cancer, relying on this approach may reduce the number of confirmed cases. Second, the results of this study were derived from an analysis of data from the USA, and so further validation is needed to determine whether the results are applicable to other countries. Third, the specific reasons for the change in survival rates, such as improved survival, were not identified in this study, and it is remains unclear whether advances in treatment or improvements in early diagnosis reduced mortality or prolonged patient survival. Fourth, this study lacked information on treatment techniques, serum LDH, and ECOG fitness status. Fifth, it is noteworthy that this study predicted a decrease in relative survival among American Indians/Alaskan Natives during 2016–2020. However, the SEER database has a relatively large proportion of white patients, and the results of the analysis of American Indians/Alaskan Natives should be interpreted with caution due to the smallness of the sample.

## Conclusion

In conclusion, according to registry data from the SEER database, the relative survival rate of patients with NHL improved gradually between 2004 and 2018, and this trend was predicted to continue during 2019–2023. This progress can be linked to the efficacy of new treatment choices, as well as improvements in risk factors due to factors such as the environment and diagnostic procedures. However, there are differences in survival rates between older and younger adults. As the population ages and the average age of patients with cancer increases, there is an urgent need to improve NHL treatments, including reduce their toxic effects on older patients. This study also found that survival rates were higher in females, that survival differences between races decreased over time, and that EN-NHL had a better prognosis than N-NHL, suggesting that primary site was not a significant factor in the prognosis.

## Data availability statement

The raw data supporting the conclusions of this article will be made available by the authors, without undue reservation.

## Author contributions

SX: Formal analysis, visualization, writing – original draft, writing – review &editing. ZY: Data curation, writing – original draft, writing – review & editing. AF: Data curation, formal analysis, writing – review & editing. SZ: Data curation, formal analysis, writing – review & editing. YL: Writing – original draft, writing – review & editing. YZ: writing – original draft, writing – review & editing. JL: Visualization, writing – original draft, writing – review & editing. All authors contributed to the article and approved the submitted version.

## Funding

The study was supported by Guangdong Provincial Key Laboratory of Traditional Chinese Medicine Informatization (2021B1212040007).

## Conflict of interest

The authors declare that the research was conducted in the absence of any commercial or financial relationships that could be construed as a potential conflict of interest.

## Publisher’s note

All claims expressed in this article are solely those of the authors and do not necessarily represent those of their affiliated organizations, or those of the publisher, the editors and the reviewers. Any product that may be evaluated in this article, or claim that may be made by its manufacturer, is not guaranteed or endorsed by the publisher.
